# Hereditary hemorrhagic telangiectasia- a new surgycal approach

**DOI:** 10.1016/S1808-8694(15)31138-1

**Published:** 2015-10-20

**Authors:** Diego Rosado de Miranda, Márcio Meira Lima, André Luiz Monteiro Cavalcante, Elias Bezerra Leite, Sebastião Diógenes Pinheiro, Marcos Rabelo de Freitas

**Affiliations:** 1 Graduated from the UFRN-2000. ENT Resident-UFC; 2 MD. Graduated from UFC. ENT Resident -UFC; 3 ENT - UFC, Otorhinolaryngologist – Department of otorhinolaryngology – Federal University of Ceará (UFC); 4 ENT - UFC. Otorhinolaryngologist – Department of otorhinolaryngology – Federal University of Ceará; 5 PhD in Medicine – University of São Paulo. Adjunct Professor of Otorhinolaryngologist – UFC Medical School; 6 M.S. in Medicine - USP/ Ribeirão Preto. Assistant Professor of Otorhinolaryngology UFC

**Keywords:** epistaxis, rendu-osler-weber, telangiectasia

## INTRODUCTION

Hereditary Hemorrhagic telangiectasia (HHT), or Rendu-Osler-Weber disease, is a vascular anomaly characterized by multiple dilations of skin and mucosa capillaries and venules. It is an autosomal dominant disease, equally distributed between both genders and its incidence is of 1-2/100.000 inhabitants.1

Bleeding may occur in numerous places; however, epistaxis is the most common, present in 90% of the cases^2^. The basic lesion is found on the vessels' walls, with defects in the elastic and muscular layers that make them more prone to bleeding^1^,^3^.

Many treatment modalities have been used to control epistaxis; none of them have rendered entirely satisfactory result^4^, ^5^. Options are nasal packing, hormone-therapy, vascular embolizations, fascioplasty and septodermoplasty. Young's surgery is based on nasal occlusion, thus preventing the friction between air and the telangiectasia, precluding epistaxis episodes.^6^

## CASE REPORT

**Figure 1:**
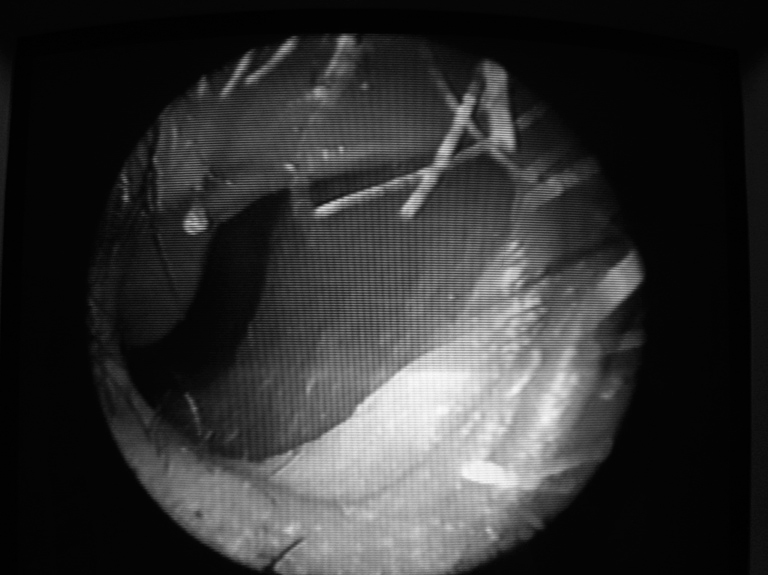
skin-mucosa joint in the nasal cavity: incision site and closure in two suture planes - Young's approach.

A.B.L., 56 years old, history of recurrent epistaxis, mainly affecting the left side, since he was 15 years of age. He had been submitted to many blood transfusions, as well as cauterizations and fascioplasty, with less than satisfactory results. At hospital admittance he had lost much weight and had excessively pale skin (+++/4+). Multiple telangiectasia lesions were seen on the upper lip, nasal tip and nasal cavities. His hematocrit count was 24%. The patient was then submitted to Young's procedure on the left side, since this one was the most symptomatic one. A circular incision was carried out on the skin-mucous joint (Figure 1), preserving the vibrissae on the side of the nostril, and suturing up in two planes, a more internal mucous layer and another external, skin layer. After two years and three months of follow up there was no other epistaxis episode and he also had hematologic normalization. There was no respiratory disorder and the cosmetic aspect was very satisfactory.

## DISCUSSION

Repetition epistaxis is the most common HHT symptom, present in 90% of the cases^2^. This was the only symptom our patient had. The condition started on his second decade of life, becoming increasingly more intense, in agreement with data from the literature.

The effectiveness of Young's technique in controlling epistaxis in patients with HHT is due to terminating with the air turbulence in a frail mucosa bearing diseased vassels^6^. This procedure is not much reported in the literature, and it is extremely efficient in controlling recurrent epistaxis. Our patient did not have any epistaxis episode after two years and four months of surgery, and such time is in agreement with data presented in the literature.

## FINAL REMARKS

Young's procedure proved to be efficient in controlling recurrent epistaxis secondary to HHT. We did not observe any respiratory alteration, and nasal occlusion was well tolerated. Although the number of patients submitted to this technique is still small, we may state that these procedures may be included in the therapeutic weaponry of our specialty in an attempt to minimize the symptoms of these patients, and thus improve their life quality.
